# Sex-Specific Expression of Non-Coding RNA Fragments in Frontal Cortex, Hippocampus and Cerebellum of Rats

**DOI:** 10.3390/epigenomes6020011

**Published:** 2022-04-02

**Authors:** Anna Fiselier, Boseon Byeon, Yaroslav Ilnytskyy, Igor Kovalchuk, Olga Kovalchuk

**Affiliations:** 1 Cumming School of Medicine, University of Calgary, Calgary, AB T2N 1N4, Canada; anna.fiselier@gmail.com; 2 Biomedical and Health Informatics, Computer Science Department, State University of New York, 2 S Clinton St, Syracuse, NY 13202, USA; boseon.byeon@oswego.edu; 3 Department of Biological Sciences, University of Lethbridge, Lethbridge, AB T1K 3M4, Canada; slava.ilyntskyy@uleth.ca

**Keywords:** non-coding RNA, ncRNA fragments, tRFs, frontal cortex, hippocampus, cerebellum, sex-specific

## Abstract

Non-coding RNA fragments (ncRFs) are processed from various non-coding RNAs (ncRNAs), with the most abundant being those produced from tRNAs. ncRFs were reported in many animal and plant species. Many ncRFs exhibit tissue specificity or/and are affected by stress. There is, however, only a handful of reports that describe differential expression of ncRFs in the brain regions. In this work, we analyzed the abundance of ncRFs processed from four major ncRNAs, including tRNA (tRFs), snoRNA (snoRFs), snRNA (snRFs), and rRNA (rRFs) in the frontal cortex (FC), hippocampus (HIP), and cerebellum (CER) of male and female rats. We found brain-specific and sex-specific differences. Reads mapping to lincRNAs were significantly larger in CER as compared to HIP and CER, while those mapping to snRNAs and tRNA were smaller in HIP than in FC and CER. tRF reads were the most abundant among all ncRF reads, and FC had more reads than HIP and CER. Reads mapping to antisense ncRNAs were significantly larger in females than in males in FC. Additionally, males consistently had more tRF, snRF, and snoRF reads in all brain regions. rRFs were more abundant in males in FC and females in HIP. Several tRFs were significantly underrepresented, including tRF-ValCAC, tRF-ValACC, and tRF-LysCTT in all brain regions. We also found brain- and sex-specific differences in the number of brain function-related mRNA targets. To summarize, we found sex-specific differences in the expression of several ncRNA fragments in various brain regions of healthy rats.

## 1. Introduction

Non-coding RNAs (ncRNAs) represent a large category of RNA molecules that do not encode a protein. The group consists of well-known molecules such as tRNAs, rRNAs, snoRNAs, snRNAs, as well as micro-RNAs (miRNAs), long ncRNAs (lncRNAs), small interfering RNAs (siRNAs), and many more classes and subclasses.

miRNAs and lncRNAs are differentially expressed in different organs and in response to different environmental stimuli, disease, or aging [1]. Sex-specific differences in the expression of microRNAs (miRNAs) [2,3,4] and long non-coding RNAs (lnc-RNAs) [5,6] in the brain of mammals in health and disease are also well-documented.

The information about the tissue-specificity of expression of tRNAs, rRNAs, snoRNAs, and snRNAs is less complete. For example, it was shown that tRNA expression exhibits tissue-specificity in mammals, although the overall anticodon pool of each tRNA family is similar across tissues [7]. Similarly, rRNA and their variants [8] as well as snoRNAs [4,9] and snRNAs [4,10] exhibit tissue-specific expression.

Regarding the brain-specific expression of ncRNAs, using 11 mice tissues, it was recently shown that the brain contains the largest number (~400) of unique small ncRNA transcripts [4]. Nearly 100 of these were miRNAs, including well-described brain-specific miRNAs, such as mir-9, mir-124, mir-219, mir-338 as well as novel miRNAs, such as mir-666, mir878, and mir-433. This study also found eSnora35 (MBI-36) and Snord116 (MBII-85), known to be involved in neurodevelopmental disorders, to be brain exclusive. Unfortunately, the authors used whole-brain tissues, so it is unclear whether there are region-specific ncRNAs or not.

Differential expression of miRNA families was observed between frontal cortex (FC) and hippocampus (HIP) brain regions; specifically, the miR-8 family, miR-182/miR-96/miR-183 cluster, and miR-212/miR-312 cluster were overexpressed in FC and miR-34 family overexpressed in HIP [11].

Many ncRNAs are further processed into fragments, known as ncRNA fragments (ncRFs). For a long time, they were considered degradation products. However, the discoveries of the recent decade demonstrate that ncRFs are involved in many aspects of the physiology of various organisms and are also implemented in response to stress.

Most versatile fragments are produced by processing tRNAs and are named tRNA fragments or tRFs; such molecules are called tRNA-derived small RNAs (tsRNAs) and include 5′ and 3′ tRNA halves (5′tRHs and 3′tRHs, also referred to as 5′tRF and 3′tRF), 1-tRF, 5′ and 3′ tiRNAs. These molecules are the result of processing by different mechanisms/factors. 5′ tRH molecules are produced by endonucleolytic cleavage and exonuclease digestion in the D-loop, while 3′ tRH are the result of the cleavage in the T-loop [12]. The 1-tRF series is produced by RNase Z (or ELAC2) cleavage of the pre-tRNA during the tRNA processing. Mature tRNA can be cleaved in the anticodon loop by ANG to produce 5′-tiRNA and 3′-tiRNA series under stress conditions [12]. In some experiments, up to a quarter of all ncRNA reads are represented by tsRNA molecules [4].

tsRNAs were found to regulate many cellular processes, including protein translation [13,14] and cell proliferation [15]. They are also involved in the defense response mechanisms in various organisms, including *Escherichia coli* defense against bacteriophages [16] and human cells against trypanosoma [17] or viruses [18]. In addition, tsRNAs were also shown to initiate the reverse transcription and inhibit the activation of retroviruses and retrotransposons [19,20,21]. Furthermore, tsRNAs are associated with diseases such as cancer [22,23] and amyotrophic lateral sclerosis [24]. Finally, tRNA fragments were shown to play an important role in transgenerational memory leading to the appearance of various metabolic disorders and addictive behavior in mice [25,26,27] and differential stress response in plants [28].

Subsequent analysis also revealed tissue-specific differences in the processing of ncRFs in many organisms, from plants [29] to mammals [4]. In addition, tissue-specific differences in relative abundance in tRF types were found for both nuclear and mitochondrial fractions of tRFs [4].

Information on differential expression of ncRFs in different brain regions is scarce. We only found one report on the analysis of various tsRNAs in the hippocampus of various primates [30]. The authors identified similarities in the abundance of several major miRNAs and tsRNAs and proposed several targeting rules for these molecules. Unfortunately, no comparison to other brain regions was made.

To date, there has been no comprehensive study completed on the abundance of various ncRFs in different brain regions. Moreover, no comparison was made between males and females. Therefore, in this work, we analyzed ncRNA sequencing data from the hippocampus, frontal cortex, and cerebellum of male and female rats.

## 2. Results

### 2.1. Comparison of ncRNA Abundance between Different Brain Regions in Male and Female Rats

The total number of reads ranged from 1,310,154 to 4,344,660. We first analyzed the mapping of reads to rat genome. The percentage of mapped sequences ranged from 88.70% to 97.19%, with no significant difference between the brain regions of male vs. female (Table S1). In this work, we combined the sequencing data from biological repeats into one sample; this was undertaken to increase the number of reads for rare reads mapping to ncRFs, especially in those cases where the processing from ncRNAs was rare. There was little variation in quality of reads or distribution of any specific ncRNA group among biological repeats; thus pooling sequence data together did not affect the quality of the analysis.

**Figure 1:**
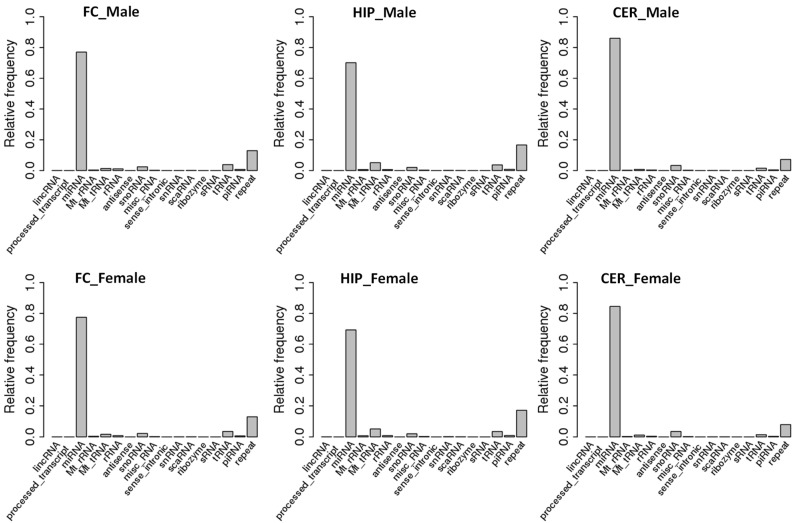
Mapping of reads to various ncRNAs in FC, HIP, and CER of male and female rats. The Y-axis shows the mapping out of 1.0, representing 100%. The X-axis shows various types of ncRNAs.

We then analyzed the distribution of ncRNA read size and found no difference between different brain parts or between males and females—22 nt was a predominant fraction in all regions of the brain in both sexes (Figure S1). This is likely because reads predominantly mapped to miRNAs, which are ~22 nt in size on average (Figure 1). A comparison of different brain regions showed that the smallest fraction of miRNAs was in HIP, while the largest was in CER. Specifically, miRNA fractions in males were ~77%, 70%, and 83% in FC, HIP, and CER, respectively; it was similar in females ~77%, 68%, and 84% in FC, HIP, and CER, respectively. The second-largest fraction of ncRNA reads was reads associated with repetitive elements. Fractions of reads associated with tRNA, rRNA, snoRNA, and snRNA were smaller than 2–3%.

### 2.2. Comparison of Average Read Size for Various ncRNA Classes between Brain Regions of Male and Female Rats

**Figure 2:**
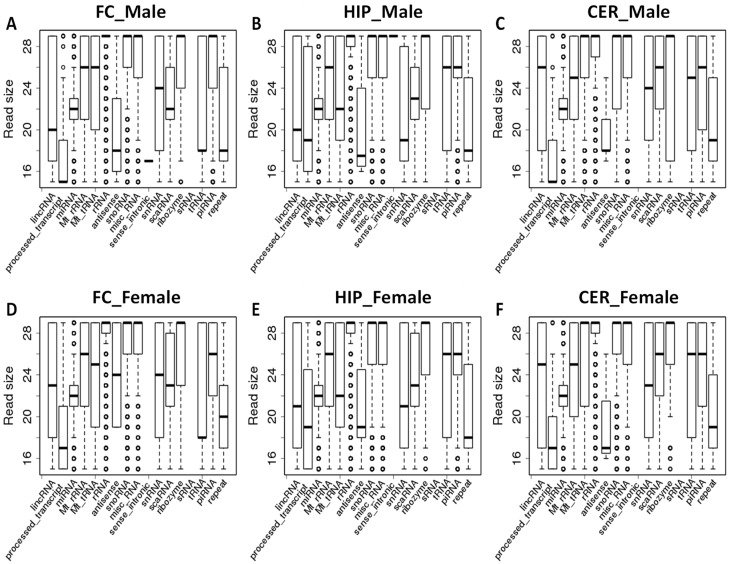
Size distribution of reads mapping to different ncRNAs in brain regions of male and female rats. (**A**) “FC_Male”—frontal cortex of male rats; (**B**) “HIP_Male”—hippocampus of male; (**C**) “CER_Male”—cerebellum of male; (**D**) “FC_Female”—frontal cortex of female rats; (**E**) “HIP_Female”—hippocampus of female; (**F**) “CER_Female”—cerebellum of female. The Y-axis shows the size of the reads, while the X-axis shows various types of ncRNAs. The bottom and top of the rectangle indicate the first and third quartiles, respectively. The lower and upper ends of the vertical line extending outside the rectangle represent the minimum and maximum, respectively. The thick horizontal line inside the rectangle is the median, and the circle beyond the rectangle displays an outlier.

We then analyzed the read size distribution among different classes of ncRNAs. The differences in the median size of reads were observed in those mapping to lincRNAs, mt-RNAs, snRNAs, antisense ncRNAs, and tRNAs. Specifically, lincRNAs reads were comparable in size between FC and HIP—~20 nt, whereas the size of lincRNA reads in CER was significantly larger than in FC or HIP—~26 nt in males (*p* < 0.05 for both); the differences between male and female were not significant (Figure 2). Reads mapping to snRNAs in HIP of both males and females were 19–21 nt, whereas in FC and CER they were significantly different, with the median of 24–25 nt (*p* < 0.05 for both). Reads mapping to antisense ncRNAs were significantly larger in females than in males in FC (*p* < 0.05). The median size of reads mapping to tRNA was 18 nt in FC of both males and females, significantly smaller than the median size in HIP and CER of 26 nt (*p* < 0.05 in all cases). No significant difference was observed in reads mapping to processed_transcript, miRNA, Mt_rRNA, rRNA, snoRNA, miscRNA, ribozyme, scaRNA, piRNA, and repeat-associated ncRNAs.

### 2.3. Analysis of GC Content in ncRNA Reads

Analyzing the GC content of ncRNAs often gives the first approximation of potential differences in their nucleotide composition. We calculated the GC content of ncRNA reads by dividing the G and C count by the total nucleotide count in ncRNA reads. Not many differences were found in GC content of most reads, but the GC content of reads mapping to antisense ncRNAs in females differed from those in males; in males ~55–60% in all three brain regions tested, whereas in females ~32% in FC, ~55% in HIP, and ~42% in CER (Figure S2). The difference in GC content in these ncRNAs in FC and CER may likely indicate the sex-specific difference in the composition of these ncRNAs and in their function.

### 2.4. Analysis of the Distribution of Reads across the Entire Length of ncRNAs

Previous reports demonstrated that many ncRNAs are processed into fragments in an end-specific manner, either from the 5′ or 3′ end [31]. Analysis of read distribution across the whole length of ncRNAs showed bias toward the 5′ end for tRNA and repeat ncRNA as well as bias toward the 3′ end for snRNA, snoRNA, and rRNA (Figure S3). No difference between different brain regions or between males and females was found.

### 2.5. ncRF Analysis

ncRNAs can be processed into ncRFs of various sizes. Therefore, we next analyzed the ncRF abundance. Even though miRNAs were the most abundant among all ncRNAs, they are not processed into ncRFs; thus, we did not analyze this category of ncRNAs further. To compare the number of ncRF reads between different brain regions in males and females, the ncRF read numbers were prorated to reads in male frontal cortex (FC_M).

tRF reads were the most abundant among the four ncRF read types analyzed, representing 83.6–98.1% of all reads, with FC having the most tRF reads and CER having the smallest percentage (Table 1). In addition, we found that CER and HIP had lower numbers of tRF reads in both males and females, with only CER being significantly different (*p* < 0.05). Additionally, males consistently had more tRF reads in all brain regions, with FC being significantly different (*p* < 0.05).

rRF reads were less abundant in CER as compared to other regions in both males and females. In the HIP, rRFs were significantly more abundant than in FC and CER in females and significantly less abundant as compared to FC in males. Comparison of reads between males and females showed a significantly higher rRF number in FC in males and a significantly lower number in HIP in males.

snRF reads were highly abundant in HIP in both males and females and CER in females (*p* < 0.05) compared to other brain regions. Comparison of males to females showed a 3.56-fold higher number of reads in FC (*p* < 0.05).

snoRF were the second most abundant ncRF reads. Opposite to tRF and rRF, snoRFs were the most abundant in the CER in males and females. No significant difference was found between males and females.

To compare the read number between different regions in male and female, we corrected the read number by prorating all read numbers to the read number in FC, taken as “1”. FC_M, CER_M, and HIP_M show the ratio of reads in a specific region in males prorated to FC. FC_F, CER_F, and HIP_F show the same for females.

### 2.6. Analysis of ncRF Fractions and ncRF Types

**Figure 3:**
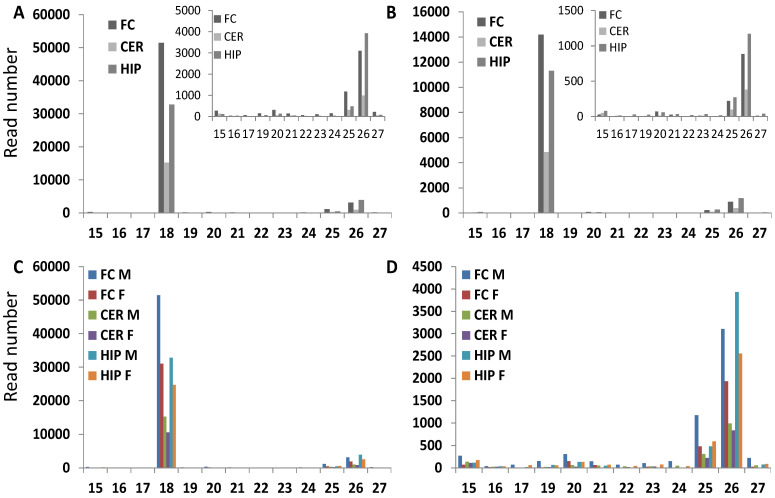
Comparison of tRF read number for tRFs of various sizes. Comparison of read number between different brain regions in male (**A**) and female (**B**). Since 18 nt reads were predominant, we generated another figure omitting 18 nt reads—see the inserts. Comparison of read number between male and female for all nt distributions (**C**) and for all but the 18 nt fraction (**D**). The Y-axis shows the number of reads for each specific group. The X-axis shows the size of reads.

We observed various fractions of tRFs in different brain regions, with the predominant fraction of tRF reads being the 18 nt fraction, followed by the 26 nt fraction. The analysis of the number of tRF reads in the most abundant fractions of 18 and 26 nt showed fewer reads in CER in both males and females; tRF reads in FC were the most abundant, followed by HIP. Comparison between males and females showed that tRF reads were more abundant in males as compared to females in all brain regions, with differences in FC and HIP being more pronounced (Figure 3). No difference between 18 and 26 nt fractions in terms of abundance in different brain areas or in males versus females was observed.

Analysis of rRFs processing revealed that they were processed from 5_8S_rRNA, Rn5–8s and 5S_rRNA. No difference was found in rRF between different tissues of males and females; rRF-3′ were of 17 nt, whereas rRF-5′ mainly were 25 nt in size (data not shown).

Analysis of snRF showed that those mapping to U1 snRNA were predominant, larger in size (17–19 nt) on average, and more diverse, whereas those mapping to U5 were a minor fraction and mostly 15 nt in size (data not shown). No significant difference between brain regions or male/female were found.

Analysis of snoRFs showed that snoRF-3′ reads were predominant in all brain regions of males and females and the ratio of snoRF-3′ to snoRF-5′ varied from ~7:1 in FC in males and in HIP in females to ~13:1 in FC in females (Figure S4).

Analysis of snoRF-3 reads showed two major fractions—15–16 nt and 25–26 nt. No significant differences were found in these fractions between brain regions in males or females (Figure S5A,B). snoRF-5 had three major fractions, 15–16 nt, 21–22 nt, and 25–26 nt. A significant difference was found between CER and other regions in small 15 nt fraction in males and between FC and other regions in females in all fractions (Figure S5C,D).

Comparisons between males and females showed significant differences in snoRF-5 in FC, especially noticeable in the largest 26 nt fraction; the 26 nt fraction of male snoRF-5 was 27%, while female fraction was 0% (Figure S6). Differences in the other regions in snoRF-5 and snoRF-3 were not significant.

### 2.7. Correlation between ncRNA Reads and ncRF Reads

**Figure 4:**
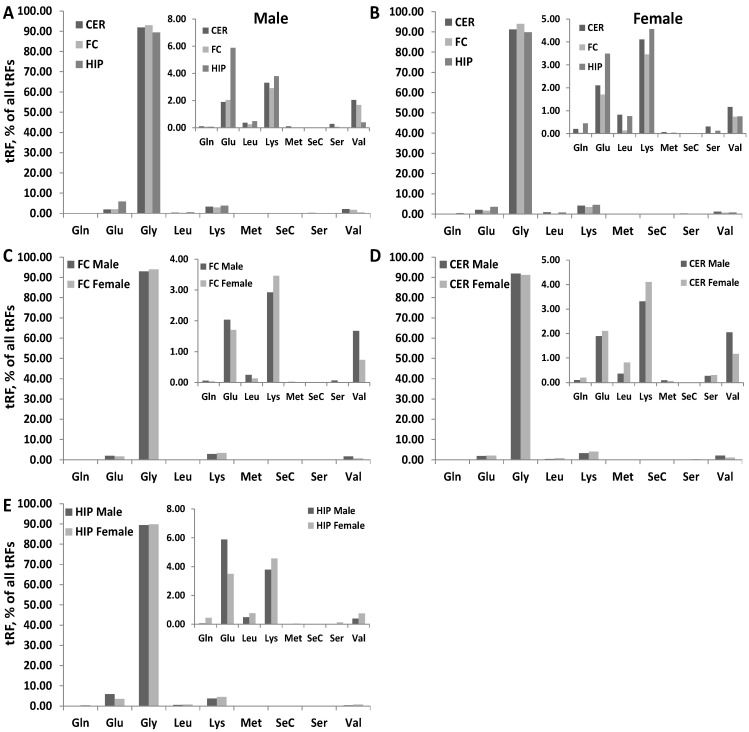
Analysis of tRF by their mapping to different tRNAs. Comparison of percentage of reads mapping to different tRNAs between different brain regions in male (**A**) and female (**B**). Since reads mapping to Gly were predominant, we generated another figure omitting Gly reads—see the inserts. (**C**–**E**) show male to female comparison for FC, CER and HIP brain regions, respectively. The Y-axis shows the percentage of reads for each specific group. The X-axis shows the tRNA the reads mapped to.

Analysis of tRFs by the type of tRNA they were processed from showed that tRF-Gly reads were the most predominant, representing ~90% of tRF reads (Figure 4); no difference was found in the percentage of tRF-Gly reads between males and females or different brain regions. The second and third largest fractions were tRF-Glu and tRF-Lys, and there were differences found in those fractions, where tRF-Glu was more abundant in HIP of males and females compared to other brain regions, with males having nearly two-fold larger tRF-Glu fraction compared to females.

**Figure 5:**
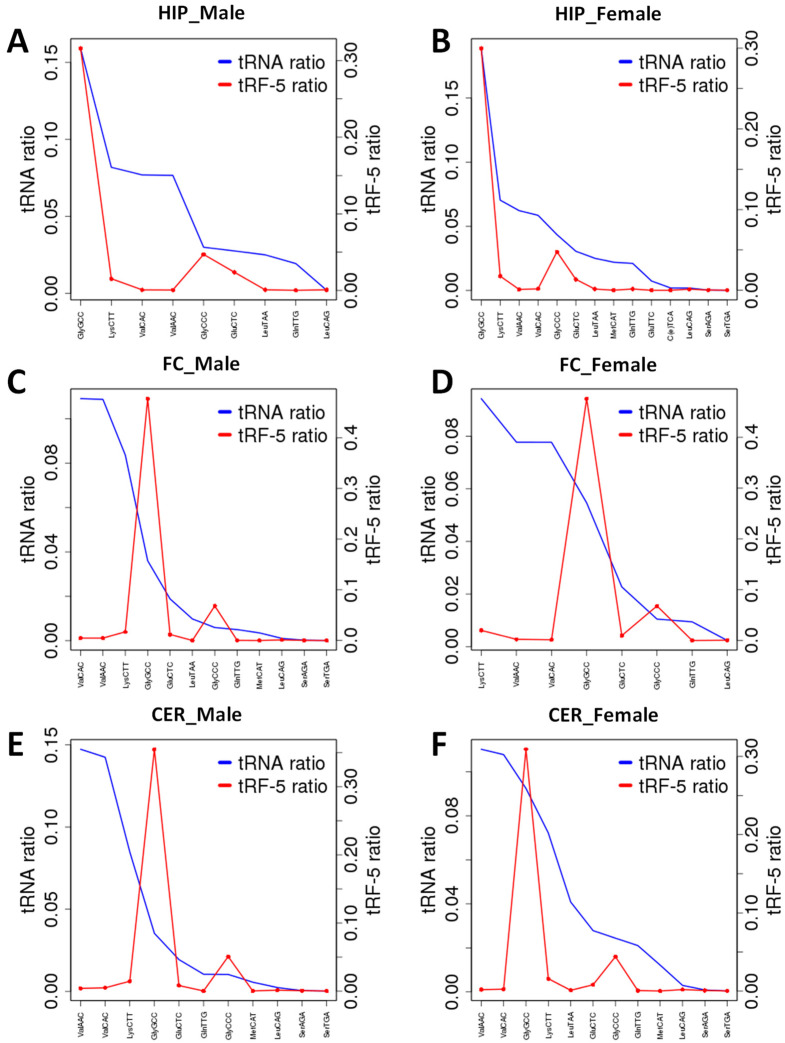
Enrichment of tRFs produced from tRNAs in various brain regions. (**A**) “FC_Male”—frontal cortex of male rats; (**B**) “HIP_Male”—hippocampus of male; (**C**) “CER_Male”—cerebellum of male; (**D**) “FC_Female”—frontal cortex of female rats; (**E**) “HIP_Female”—hippocampus of female; (**F**) “CER_Female”—cerebellum of female. The Y-axis shows specific tRNA and tRF-5 ratios for specific tRNAs and t-RFs relative to all. When the t-RF peak is larger than the tRNA peak, there is an enrichment, while when it is lower, there is underrepresentation.

RNA fragments can be produced from ncRNAs at a similar rate for all types; certain ncRNAs can be processed more frequently. We, thus, plotted reads mapping to tRF to reads mapping to tRNA (Figure 5). We found that tRF-GlyGCC was enriched in FC and CER brain regions of male and female rats; the enrichment was less pronounced in female rats. Several tRFs were significantly underrepresented, including tRF-ValCAC, tRF-ValACC, and tRF-LysCTT in all brain regions. This analysis demonstrated that certain tRNAs are indeed processed to tRFs more frequently.

**Figure 6:**
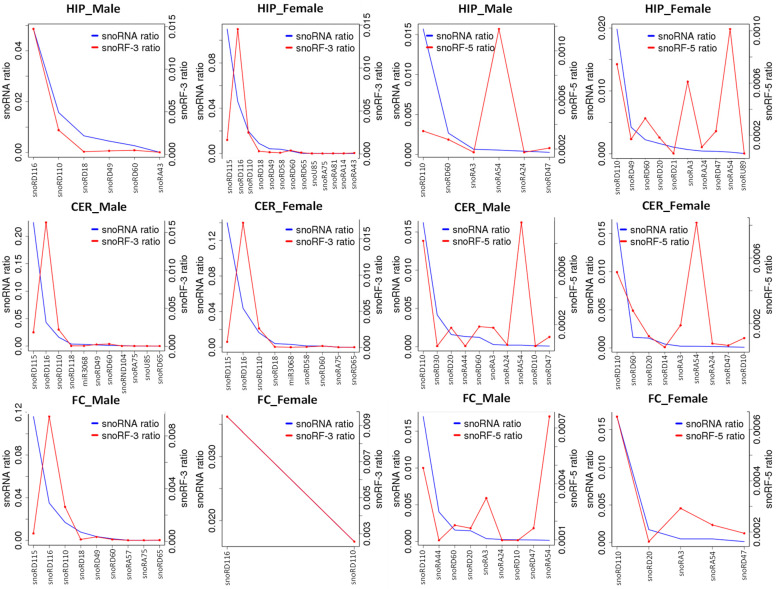
snoRF-3 and snoRF-5 enrichment from snoRFs in various brain regions. The Y-axis shows reads mapping to snoRFs or snoRF-3 and snoRF-5. When the snoRFs peak is larger than snoRNA peak, there is an enrichment, while when it is lower, there is underrepresentation.

Similar analysis of the reads mapping to snoRFs and snoRNAs showed that snoRF-3 stemming from snoRD116 were enriched in both male and female CER and FC as well as in female HIP but not in male HIP (Figure 6). Among snoRF-5 reads, those stemming from snoRA54 were enriched in all male and female brain regions, although enrichment in FC in females was less pronounced. Additionally, snoRF-5 reads mapping to snoRA-3 were enriched in female HIP and male FC. Again, this experiment showed that certain snoRNAs are processed more frequently and at different rates in different brain regions.

**Figure 7:**
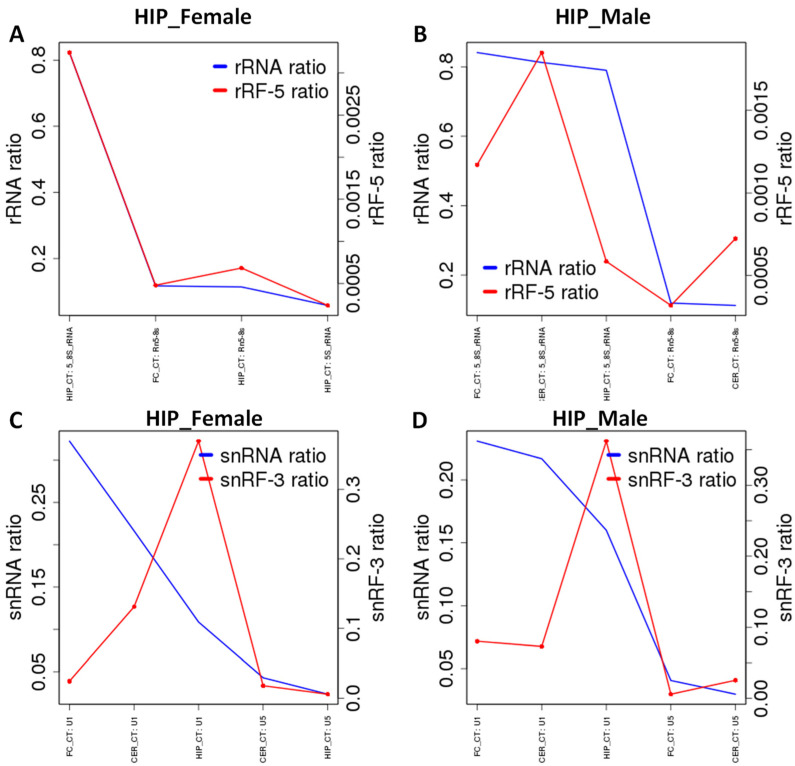
rRF and snRF enrichment from rRNA and snRNA in various brain regions. (**A**) rRNA and rRF ratios for female hippocampus; (**B**) rRNA and rRF ratios for male hippocampus; (**C**) snRNA and snRF ratios for female hippocampus; (**D**) snRNA and snRF ratios for male hippocampus. The Y-axis shows reads mapping to rRFs and snRFs or rRF-5 and snRF-3. When the rRFs and snRFs peaks are larger than rRNA and snRNA peaks, there is an enrichment, while when it is lower, there is underrepresentation.

rRF stemming from 5_8SRNA were underrepresented in male HIP. rRF stemming from U1 snRNA were enriched in female and male HIP, and underrepresented in female and male FC, although enrichment in males was less pronounced than in females (Figure 7).

### 2.8. Prediction of Targets of ncRFs in Various Brain Regions

**Figure 8:**
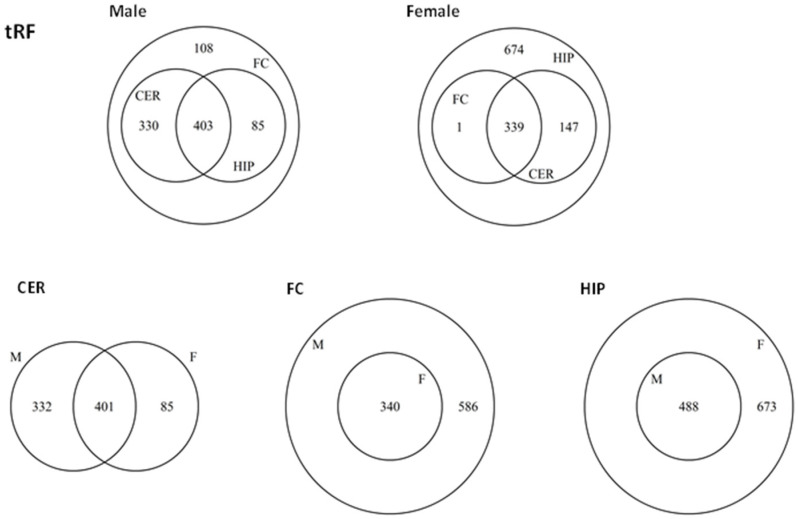
Venn diagrams of overlapping target genes (as analyzed by miRDB) of tRFs in male and female brain regions. The upper panel shows the overlap between different brain regions in male (**left** part) and female (**right** part), while the lower panel shows the overlap between male and female groups for each brain region.

We used miRDB to predict unique gene targets of ncRFs. We first compared unique and common targets for tRFs among different regions in males (Supplementary File S1). We found 926 target genes in male FC, 733 target genes in CER, and 488 in HIP; CER and HIP groups overlapped fully with the FC group (Figure 8). Comparison of male to female groups showed that for FC and HIP, there was a full overlap between males and females; in contrast, in CER, there also was a large overlap between males and females, but there were 332 unique target genes in males and 85 in females. Among uniquely targeted genes in females were PLAGL1, Tmem35, STRN3, and many other neuron-related genes (Supplementary File S1). In male CER, tRF targeted Ganglioside-induced differentiation-associated-protein 2 (Gdap2), cholinergic receptor muscarinic 1 (Chrm1), glutamate receptor metabotropic 8 (Grm8), glial cell-derived neurotrophic factor (Gdnf), glutamate receptor ionotropic delta 2 (Grid2), olfactory receptor 1587 (Olr1587), and many other brain-specific factors.

**Figure 9:**
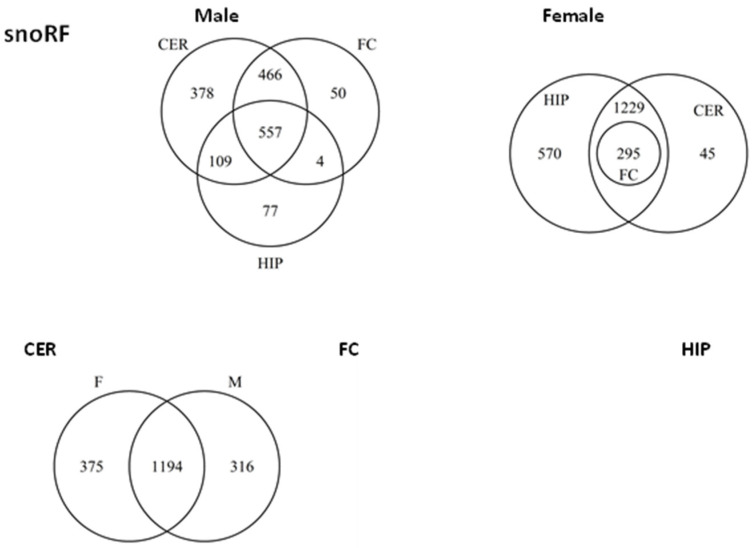
Venn diagrams of overlapping target genes (as analyzed by miRDB) of snoRFs in male and female brain regions. The upper panel shows the overlap between different brain regions in male (**left** part) and female (**right** part), while the lower panel shows the overlap between male and female groups for each brain region.

We next compared unique and common targets for snoRFs among different regions in males (Figure 9). Most of the targets overlapped between brain regions, although there were unique targets present in all regions, with the largest group being present in CER; among the unique targets were DCUN1D4, TNPO1, FUBP1, and many others (Supplementary File S1).

Comparison of unique snoRFs targets in females showed that most genes overlapped, but a large group of unique targets was identified in HIP. Comparison of targets between males and females in different brain regions showed full overlap in HIP and FC, but large sex-specific differences in CER; among targets are LPCAT1, EIF4E, and many others (Supplementary File S1).

A smaller number of targets was found for rRF and snRF, and all of them overlapped either between male and female or between different brain regions (Figures S7 and S8).

### 2.9. Analysis of Overlapping and Unique Pathways Using DAVID

**Figure 10:**
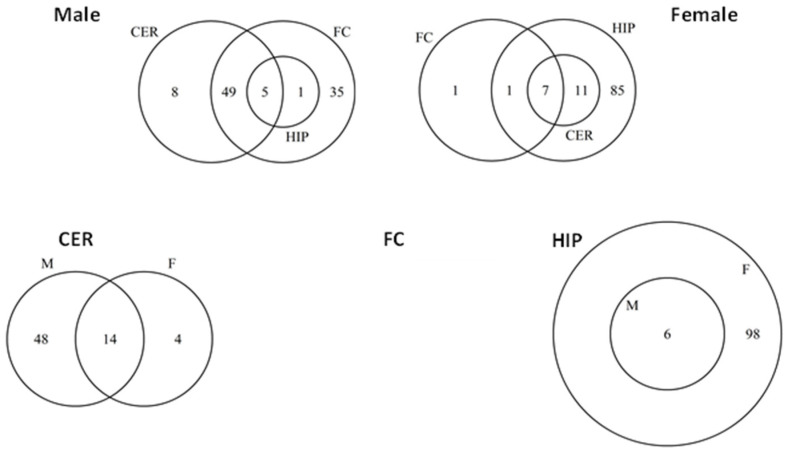
Venn diagrams of overlapping target pathways (as analyzed by DAVID) for tRFs in male and female brain regions. The upper panel shows the overlap between different brain regions in male (**left** part) and female (**right** part), while the lower panel shows the overlap between male and female groups for each brain region.

We next annotated common and unique target genes using DAVID. Significantly enriched pathways for each brain region are shown in Supplementary Files S2 and S3. Analysis of enriched pathways from tRF targets in males showed large overlaps between all groups, although FC and CER had several unique pathways (Figure 10). They included the Notch signaling pathway, ErbB signaling pathway, Ras signaling pathway, regulation of circadian rhythm, SMAD binding, and many more for FC; they also showed pathways for brain development, presynaptic membrane, and cell cycle, among others (Supplementary Files S2 and S3). Analysis of tRF targets in females showed that the HIP region has a large group of unique target pathways, including the Notch signaling pathway, MAPK signaling pathway, dendritic spine, axon guidance, among others (Figure 10).

Male to female comparison of tRF targets showed that while pathways in HIP and FC overlapped between males and females, CER had substantial differences. Females had a unique pathway-positive regulation of cell migration, while males had VEGF signaling pathway, neuron projection, brain development, dendritic spine, focal adhesion and synapse, and many more (Supplementary Files S2 and S3).

## 3. Discussion

In this work, we report the differential expression of ncRNA and ncRFs in different brain regions of male and female rats. We found tissue- and sex-specific expression, processing, 5′/3′ bias, and enrichment of various ncRFs. In Table 2, we summarize main findings of this study; we observed differences in size of various ncRNA reads and their processing (Table 2).

### 3.1. Sex-Specific Differences in ncRNA Expression

Sex-specific expression of ncRNA was observed in various tissues of both humans and animals. Simon et al. reported nine sex-specific differentially expressed miRNAs in humans [32]; moreover, they showed relevant miRNA-mRNA regulatory networks associated with sex. Similar studies in animals also showed sex-specific differences in miRNA expression. In pigs, sex-specific miRNA expression has also been reported in males and females [33]. Similar differences were observed in beetles [34]. Potential sex-specific expression patterns were also proposed for abnormal expression profiles found in The Cancer Genome Atlas database, although the differences were not apparent [35].

More data exists about sex-specific expression of long non-coding RNAs in humans. Sex-specific expression of lncRNAs also has been reported. This is interesting from a disease perspective as motor neuron diseases such as amyotrophic lateral sclerosis and X-linked spinal muscular atrophy occur more frequently in males as compared to females [36,37]. Issler et al. (2020) identified the primate-specific, neuronal-enriched gene, LINC00473, encoding lnc-LINC00473, as downregulated in the prefrontal cortex (PFC) of depressed females but not males and demonstrated that overexpression of this lnc triggers sex-specific changes in synaptic function and gene expression selectively in female mice [5].

We cannot be certain that these sex-and tissue-specific differences come from the differential expression or/and processing of ncRNAs. It is possible that male and female rats have substantially different brain region-specific composition of neurons and glia cells, accounting for the observed differences in the expression and processing of ncRNAs. Indeed, neuronal density differs between sexes; in addition, cell composition in a given brain region may also be different. For example, the hypothalamus of mice contains rare brain cell types that are unique to males or females [38]. Regardless of the causation of the differences in ncRF expression, they likely have true biological significance.

### 3.2. Brain-Specific Differences in ncRNAs and ncRFs

Our analysis showed that miRNA reads were predominant among all reads, while tRNA reads represented only ~2–3% of all ncRNA reads. In contrast, when miRNA reads were discarded, and we only analyzed the abundance of tRFs, we found that they represented 68–84% of all ncRF reads, depending on sex and brain region. In contrast, a recent analysis of ncRNA reads in different tissues of mice demonstrated that tRFs represented ~30% fraction of all reads in HIP, while only 13% in FC and CER [30]. We did not observe such drastic difference between HIP and other regions, and in contrast, in our experiments HIP had a lower percentage of tRF reads compared to the other two regions (Figure 1).

### 3.3. Difference in Processing of ncRFs

Our analysis showed a strong bias toward the 5′ end for tRNA in all brain regions, regardless of the animal’s sex. Bias in the processing of tRNAs to tRFs towards 5′ end was observed previously.

Bias towards the abundance of tRFs originating from the 5′ end is quite common. Recently, the authors observed that the distribution of nuclear tRFs was largely skewed toward 5′tRH, generated by the cleavage in the anticodon loops of mature tRNA [4]. Similarly, analysis of fractions of tRFs in different brain regions showed that over 80% were 5′tRFs [30]. Additionally, nuclear tRF fractions in the intestine were largely stemming from 5′tRH [4]. The abundance of 5′tRH fragments has been previously shown to associate with the activity of angiogenin in the cell [39]; therefore, the authors speculated that the intestine has a higher activity of Ang4 [40]. High levels of 5’ tRH were also found in the primate hippocampus [30]. In contrast, Haack et al. found a surprisingly high aggregation of 3′-tRNA halves in the amygdala and hippocampus [41].

### 3.4. Differences in the Type of tRFs

Reads mapping to tRF-Gly were a predominant fraction. Specifically, the isoacceptor tRF-GlyGCC was enriched in FC and CER brain regions of male and female rats, while isoacceptors tRF-ValCAC, tRF-ValACC, and tRF-LysCTT were underrepresented in all brain regions.

tRF-Gly was found to be the most abundant of the nuclear tRF fractions in the mouse intestine [4] and in monkey brains [30]. In addition, 5′ tRH-GlyGCC was also found to be dynamically expressed during stem cell differentiation [42]. Isoacceptor 5′ tRH-GluCTC was highly expressed in human monocytes, where it triggers the transcriptional suppression of the surface glycoprotein CD1 [43] and in the brains of several monkeys [30]. Additionally, another isoacceptor, 5′ tRH-GluTTC was enriched in the intestine [4]. The intestine was also enriched in tRF stemming from glutamine, valine, and lysine tRNAs, while 5′tRFs of proline transferring mt-Tp were abundant in the heart and 5′tRFs of asparagine transferring mt-Tn in the liver [4].

### 3.5. Predicted Targets

We used miRDB to predict potential targets of ncRFs. miRDB uses the algorithm based on targeting of mRNAs by miRNAs. There is currently no perfect software to predict targets of ncRFs. It was hypothesized that they might target mRNAs in a similar manner to miRNAs and piRNAs. It was recently shown that all 10 tested potential targets of 5′ tRH-Gly-GCC were expressed lower in the cells overexpressing this tRF as compared to controls; thus confirming that software based on miRNA/piRNA prediction likely predicts valid targets [30].

Among genes uniquely targeted by tRFs in females were PLAGL1, Tmem35, STRN3, and several other neuron-related genes. PLAGL1 is a paternally expressed imprinted gene [44], where the copy of the gene that comes from the mother is inactivated by methylation. The fact that we found tRFs that uniquely target imprinted genes may suggest that tRFs provide a second layer of protection by targeting “escape” mRNA copies for degradation. Tmem35 encodes a soluble peptide that may interact with NGFR and modulate neurite outgrowth (https://www.uniprot.org/uniprot/Q53FP2#function, accessed on 15 December 2021). TMEM35 is a novel factor required for normal activity of the HPA axis and limbic circuitry [45]. STRN3 (Striatin 3) encodes the protein functioning in glutamate regulation of dopamine D1A receptor signaling. STRN3 is a novel interaction partner of the glucocorticoid receptor (GR) that interferes with GR’s ligand-dependent transactivation capacity [46].

Among predicted tRF targets in male CER were Ganglioside-induced differentiation-associated-protein 2 (Gdap2), cholinergic receptor muscarinic 1 (Chrm1), glutamate receptor metabotropic 8 (Grm8), glial cell-derived neurotrophic factor (Gdnf), glutamate receptor ionotropic delta 2 (Grid2), olfactory receptor 1587 (Olr1587), and many other brain-specific factors.

Among unique snoRF targets in male CER were DCUN1D4, TNPO1, FUBP1, and many others (Supplementary File S1). Deficiency in DCUN1D4 (Defective In Cullin Neddylation 1 Domain Containing 4) leads to the development of optic nerve sheath meningioma. TNPO1 (Transportin 1) is involved in checkpoint regulation and CDK-mediated phosphorylation and removal of Cdc6; mutation of TNPO1 leads to retinitis pigmentosa 2 and meningoencephalitis. A recent report shows that microtubule stabilization in Hutchinson–Gilford progeria syndrome (HGPS) cells sequestered TNPO1 in the cytoplasm, affecting the nuclear localization of its cargo, including the nuclear pore protein NUP153 [47]. Consequently, nuclear Ran, nuclear anchorage of the nucleoporin TPR, and chromatin organization were disrupted, deregulating gene expression and inducing senescence.

In females, unique targets of snoRFs in CER were LPCAT1, EIF4E, and many others (Supplementary File S1). LPCAT1 encodes lysophosphatidylcholine acyltransferase 1, a protein that catalyzes the conversion of LPC to phosphatidylcholine (PC) in the remodeling pathway of PC biosynthesis. EIF4E encodes eukaryotic translation initiation factor 4E that regulates signaling mediated by p38 and pathways affecting insulin-like growth factor (IGF1)-Akt signaling. Diseases associated with EIF4E include autism and pervasive developmental disorder.

## 4. Materials and Methods

### 4.1. Animals Used in the Experiment

Tissues from control male and female three-month-old Long Evans rats (Charles River, Wilmington, MA, USA) were used in this study; three animals of each sex were used in the analysis. These animals were also used as sham-treated controls in the study aimed to establish radiation effects on the brain [48,49]. The animals were housed in a pathogen-free controlled facility with a 12 h light/dark cycle and given food and water ad libitum. The handling and care of the animals were conducted in accordance with the recommendations of the Canadian Council for Animal Care and Use. The University of Lethbridge Animal Welfare Committee approved all procedures.

### 4.2. Sequencing

The ncRNA sequencing was performed on Genome Analyzer IIx using single sequence reads of 36 nt; 36 nt reads include a 7-nt adaptor and a 29-nt sequence of ncRNA. For the analysis of sequencing data, biological repeats were pooled together.

Sequencing reads were processed from fastq format and then aligned to the downloaded ncRNA sequences using Bowtie with the options of ‘-v 2-best’. To identify a specific type of ncRNA read in different tissues, we classified all ncRNA reads and presented them as the average frequency of occurrence (as a fraction of 1).

### 4.3. The Identification and Description of ncRFs

ncRNAs matching the 5′-end and 3′-end of ncRNAs were defined as ncRNA fragments (ncRFs). We only took into consideration those reads that were smaller than 27 nt. This was done to exclude the fraction of longer reads stemming from sequences of ≥29 nt. The 29 nt fraction of reads stemmed from longer inserts of ncRNAs. In contrast, the fraction of ≤27 nt actually represented the processed fragments of ncRNAs or ncRFs. Only those ncRNAs that had ≥5 reads were included in the analysis.

### 4.4. The Analysis of the Distribution of ncRF Reads across the Entire Length of Precursor ncRNAs

Each ncRNA sequence was divided into 10 equal-sized bins, and ncRF reads in each bin were counted across all ncRNAs in each ncRNA type. The distribution of ncRF reads was calculated by dividing the read count in each bin by the total read count in all bins in each ncRNA type.

### 4.5. The Analysis of the Enrichment of ncRFs Relative to the Number of ncRNA Precursors

ncRNA reads and ncRFs were extracted for each ncRNA type. The enrichment was calculated by dividing the number of ncRF reads (those that were ≤27 nt) by the number of ncRNA reads (those that were ≥29 nt). The ratio of 1 indicates that there was one ncRF for each ncRNA.

### 4.6. ncRFs Target Prediction Using miRDB

miRDB is an online database tool for miRNA target prediction. We entered the ncRF sequences into the miRDB web interface and miRDB returned the predicted targets. To be more specific, unique ncRF sequences were extracted and targets predicted using miRDB. The unique ncRF sequences were provided to miRDB and the predicted target genes were retrieved along with their target scores. According to the miRDB website (http://www.mirdb.org/faq.html#How_to_interpret_the_target_prediction_score, accessed on 15 December 2021), the predicted targets have scores between 50 and 100 and a predicted target with a target score greater than 80 is most likely to be real [50]. Therefore, the target genes of the unique ncRF sequences with the prediction target score greater than 80 were selected from the miRDB predicted targets. Each miRDB excel file (Supplementary File S1) has three worksheets of unique ncRF sequences, targets predicted by miRDB, and unique targets. Venn diagrams for overlapping gene targets were built on the lists of unique targets using the R package VennDiagram.

### 4.7. ncRFs Target Pathway Prediction Using DAVID

The functional annotation of the unique targets predicted by miRDB was performed using DAVID. The target gene symbols were provided to the DAVID web interface and the functional annotation charts were generated. The chart displays the functional annotation of the target genes, including significant ontologies and pathways. Venn diagrams were built using groups of statistically significantly different pathways in each category. The cutoff was made at Benjamini correction less than 0.05.

## 5. Conclusions

Here we reported tissue- and sex-specific differences in the expression of ncRNAs and ncRFs. Such differences could occur due to: different composition of cells in specific brain region of male/female animal; differential expression of ncRNA coding genes or their maturation; differential activity of ncRNA biogenesis enzymes, or all three together. The exact mechanism(s) remains to be uncovered. We predicted that some of the analyzed ncRFs likely have unique targets, but it still remains to be shown how all these sex- and brain-specific differences in the expression of ncRFs and the expression of their predicted targets contribute to sex-specific difference in brain physiology, brain function, and potential for pathology and development of disease. Additionally, in this work we did not analyze variant miRNA transcripts, so called isomiRs, because they are not the result of processing of mature miRNAs. In the future, however, it would be important to analyze them, as they may also be produced in tissue- and sex-specific manners.

## Figures and Tables

**Table 1:** Prorated ncRF read number in FC, CER, and HIP of males and females.

		FC_M	CER_M	HIP_M	FC_F	CER_F	HIP_F
tRF	Reads	57,307.00	17,374.36	37,031.61	31,510.58	12,117.04	28,367.62
	% total reads	98.1	88.8	94.6	97.4	83.6	93.3
	Ratio/FC	1.00	*0.30* *	0.65	1.00	*0.38* *	0.90
	Ratio_M/F	*1.82* *	1.43	1.31			
rRF	Reads	42.00	23.18	17.39	10.19	0.00	81.74
	% total reads	7.2 × 10^−4^	1.2 × 10^−3^	4.4 × 10^−4^	3.1 × 10^−4^	0	2.7 × 10^−3^
	Ratio/FC	1.00	0.55	*0.41* *	1.00	0.00	*8.02* *
	Ratio_M/F	*4.12* *		*0.21* *			
snRF	Reads	87.00	117.33	893.96	24.46	196.79	746.73
	% total reads	1.5 × 10^−3^	6.0 × 10^−3^	2.3 × 10^−2^	7.6 × 10^−4^	1.4 × 10^−2^	2.5 × 10^−2^
	Ratio/FC	1.00	1.35	*10.28* *	1.00	*8.05* *	*30.53* *
	Ratio_M/F	*3.56* *	0.60	1.20			
snoRF	Reads	989.00	2056.82	1219.20	815.23	2178.15	1200.29
	% total reads	1.7 × 10^−2^	0.1	3.1 × 10^−2^	2.5 × 10^−2^	0.15	3.9 × 10^−2^
	Ratio/FC	1.00	*2.08* *	1.23	1.00	*2.67* *	1.47
	Ratio_M/F	1.21	0.94	1.02			
Total	Reads	58,425.00	19,571.69	39,162.16	32,360.46	14,491.98	30,396.38

* defines significance of ratios (numbers are in bold and italic).

**Table 2:** Summary of main findings.

Brain Region	HIP		CER		FC	
Sex	Male	Female	Male	Female	Male	Female
miRNA	=	=	=	=	=	=
ncRNA read size, nt						
lincRNA	20	21	26	25	20	23
snRNA	19	21	24	23	24	24
antisense	18	19	18	17	19	24
tRNA	26	26	26	26	18	18
tRF-Gly	~90%	~90%	~90%	~90%	~90%	~90%
tRF-Glu	5.8%	3.5%	1.8%	2.0%	2.0%	1.7%
tRF-Lys	3.9%	4.5%	3.3%	4.0%	2.9%	3.3%
tRF-GlyGCC	=	=	+++	++	+++	+++
tRF-ValCAC	---	---	---	---	---	---
tRF-ValACC	---	---	---	---	---	---
tRF-LysCTT	---	---	---	---	---	---
snoRD116-3′	=	+++	+++	+++	+++	+++
snoRA54-5′	+++	+++	+++	+++	+++	+
snoRA-3-5′	+++	=	=	=	+++	=
5_8SRNA	--	=	=	=	=	=
U1	++	+++	=	=	--	--

“=”—no change, or no difference between male and female mice; “+”—enrichment in ncRF processing; “-”—underrepresentation of ncRFs.

## Data Availability

All supporting information can be downloaded at Supplementary Materials.

## Supplementary Materials

The following supporting information can be downloaded at: https://www.mdpi.com/article/10.3390/epigenomes6020011/s1, Figure S1: ncRNA read size distribution; Figure S2: GC content in reads mapping to different ncRNAs in FC, HIP, and CER regions of male and female brains; Figure S3. Analysis of reads distribution across the whole length of ncRNAs; Figure S4. Distribution of snoRFs mapping to the 5′ or 3′ ends of snoRNAs; Figure S5. Comparison of snoRF mapping to the 5′ and 3′ end of snoRNAs by size of the reads; Figure S6. Comparison of snoRF-5 and snoRF-3 by size between males and females; Figure S7. Venn diagrams of overlapping target genes of rRFs in male and female brain regions; Figure S8. Venn diagrams of overlapping target genes of snRFs in male and female brain regions; File S1. Unique and common targets of ncRFs. File S2. Unique and common targets in males vs. females and between different brain regions. File S3. List of GO terms in males vs. females and in different brain regions.
