# Supplementary material for: Sex-Specific Expression of Non-Coding RNA Fragments in Frontal Cortex, Hippocampus and Cerebellum of Rats
Source: Epigenomes. 2022 Apr 2;6(2):11. doi: 10.3390/epigenomes6020011 (PMC9036230; doi:10.3390/epigenomes6020011)
Supplement: Supplementary file 1 [file epigenomes-06-00011-s001.zip › Supplementary Figures.pptx]

## Slide 1
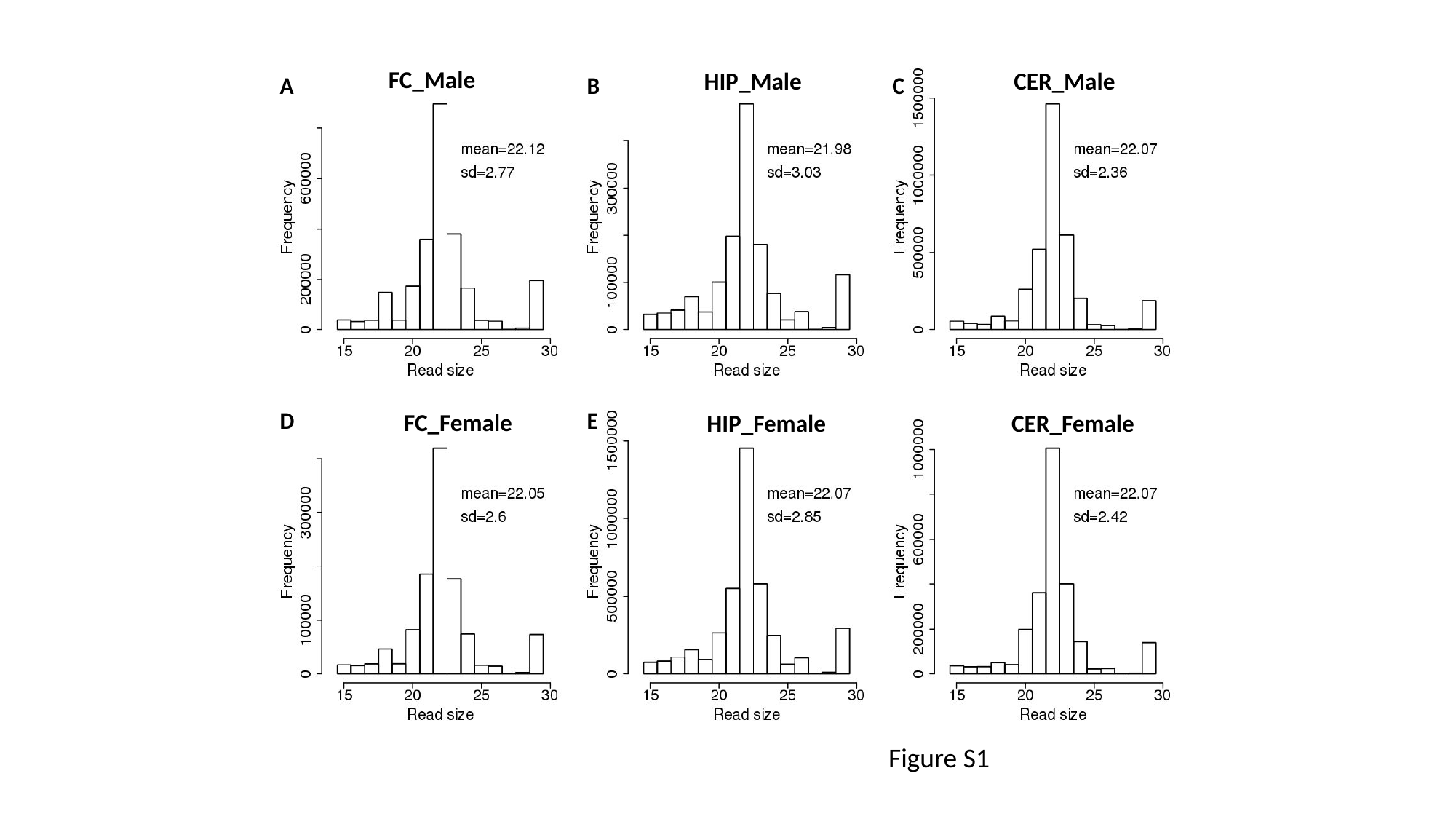

FC_Male
CER_Male
HIP_Male
FC_Female
CER_Female
HIP_Female
Figure S1

## Slide 2
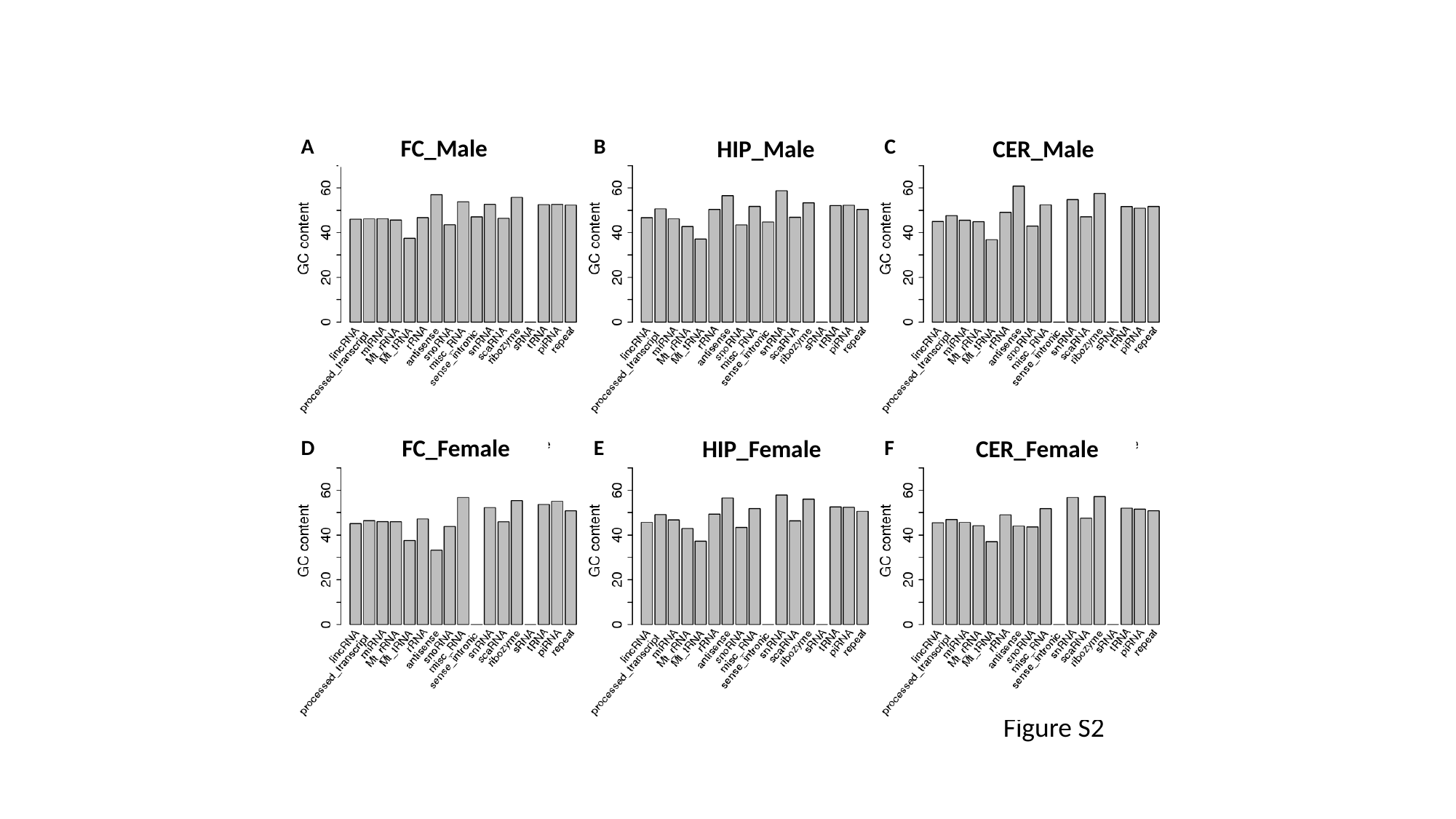

FC_Male
CER_Male
HIP_Male
FC_Female
CER_Female
HIP_Female
Figure S2

## Slide 3
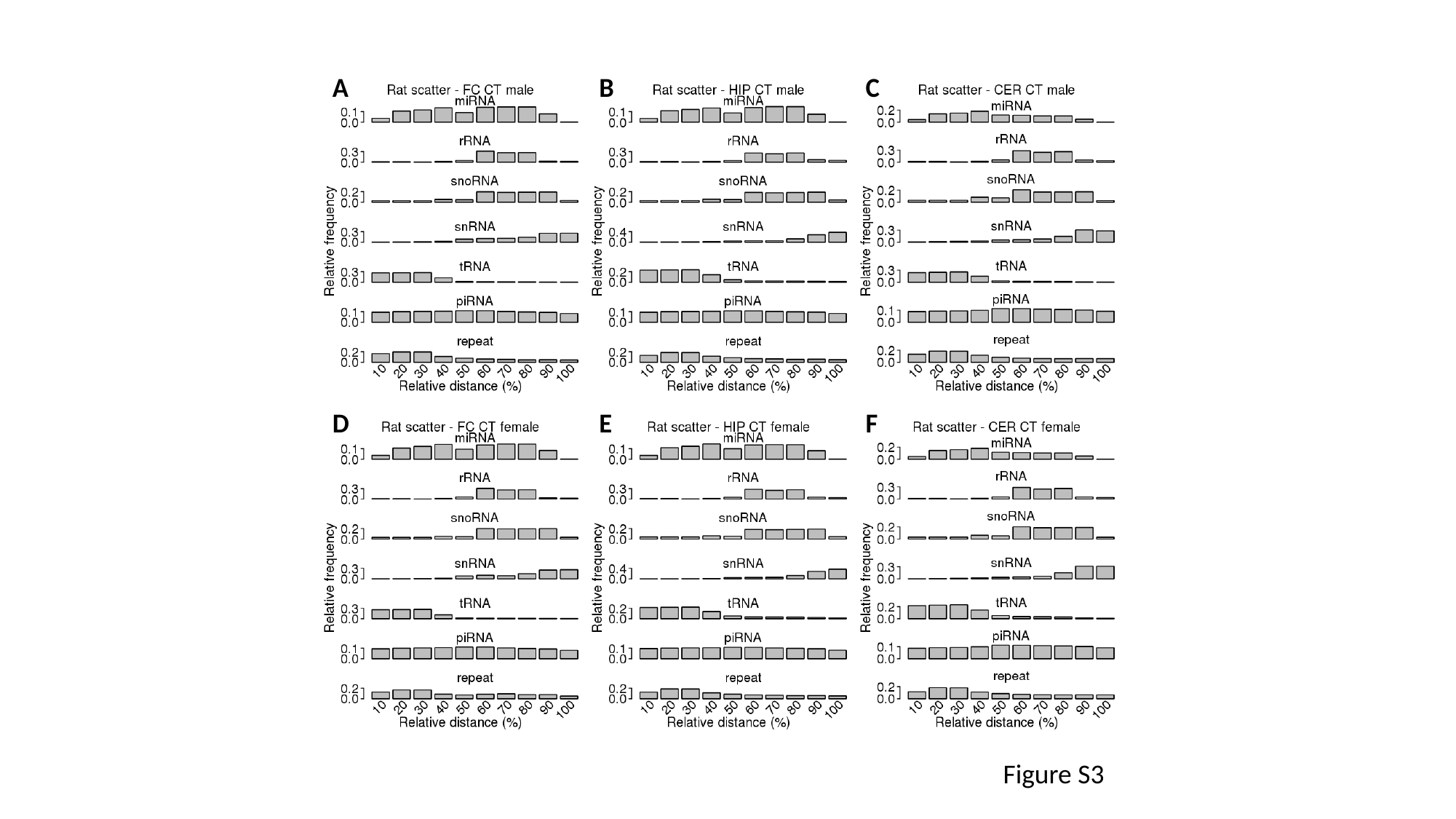

Figure S3

## Slide 4
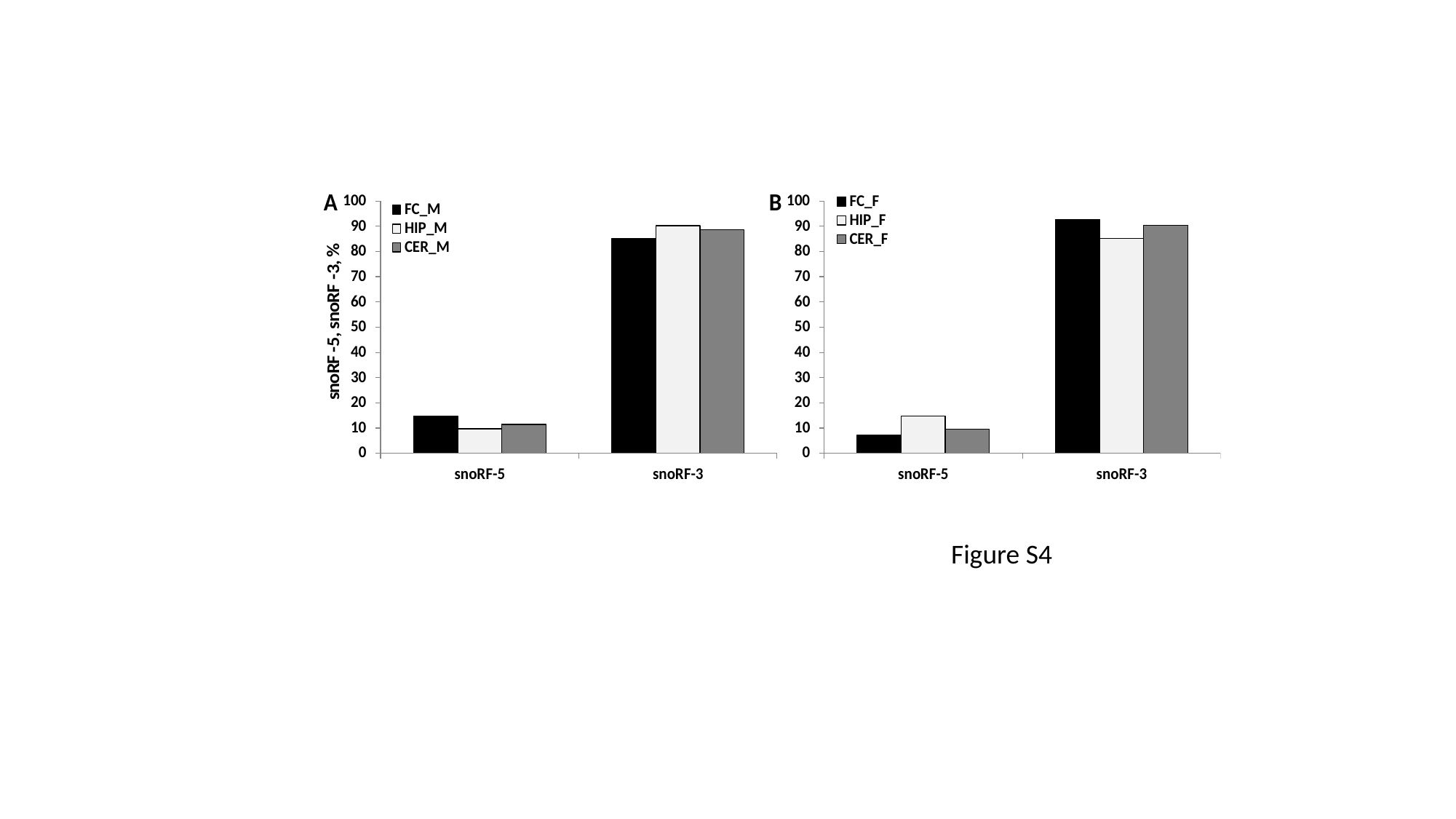

Figure S4

## Slide 5
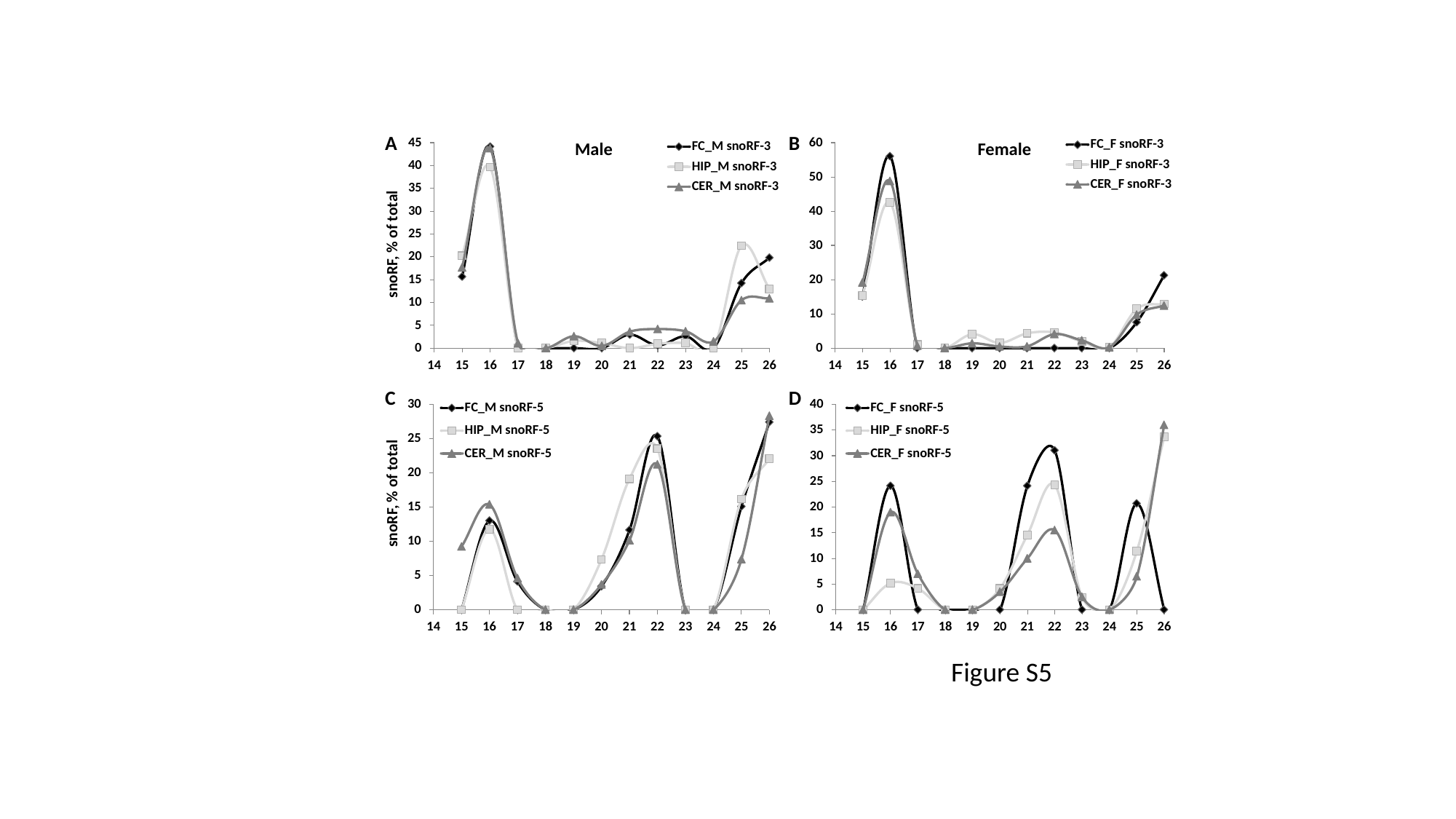

Figure S5

## Slide 6
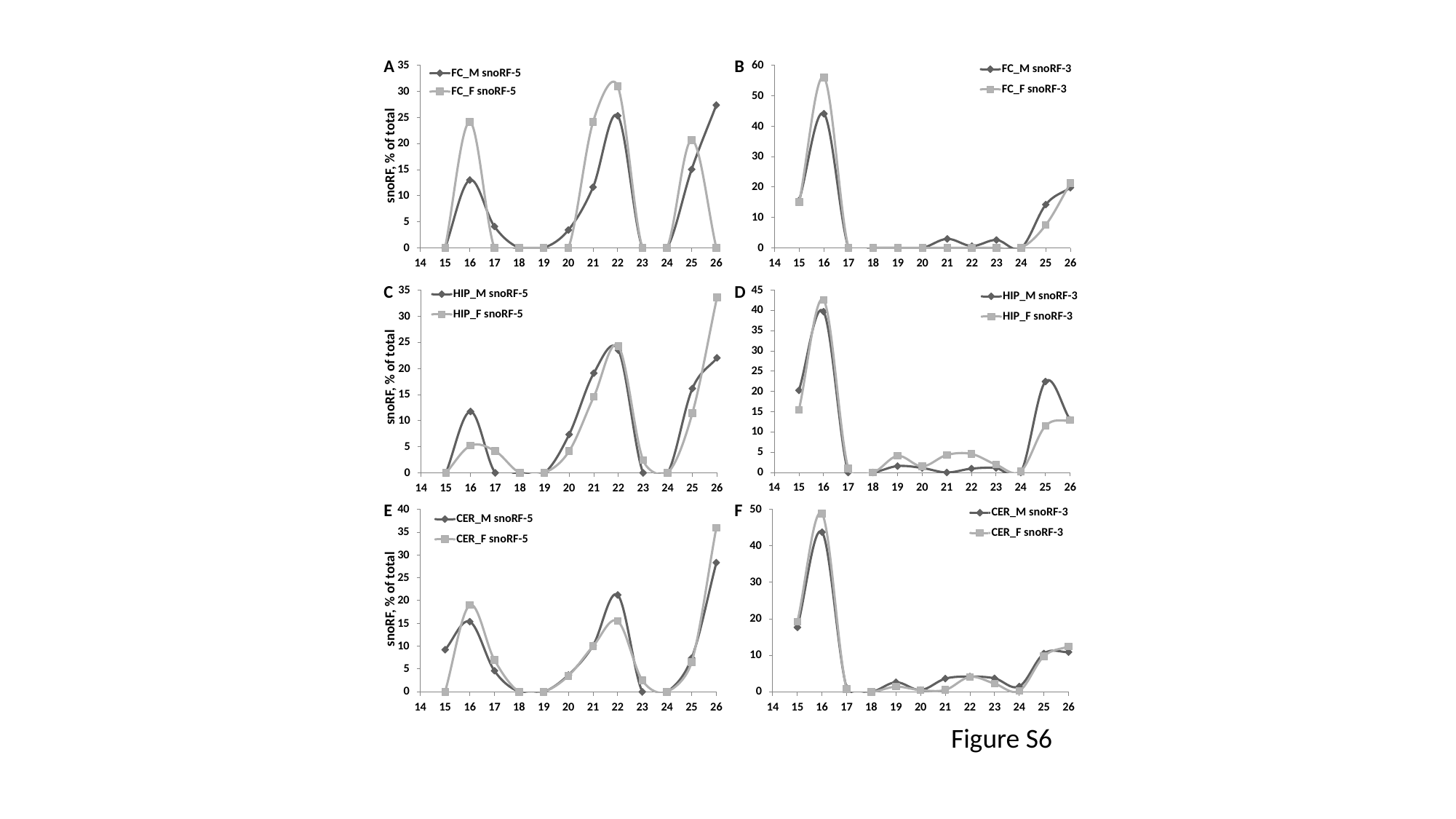

Figure S6

## Slide 7
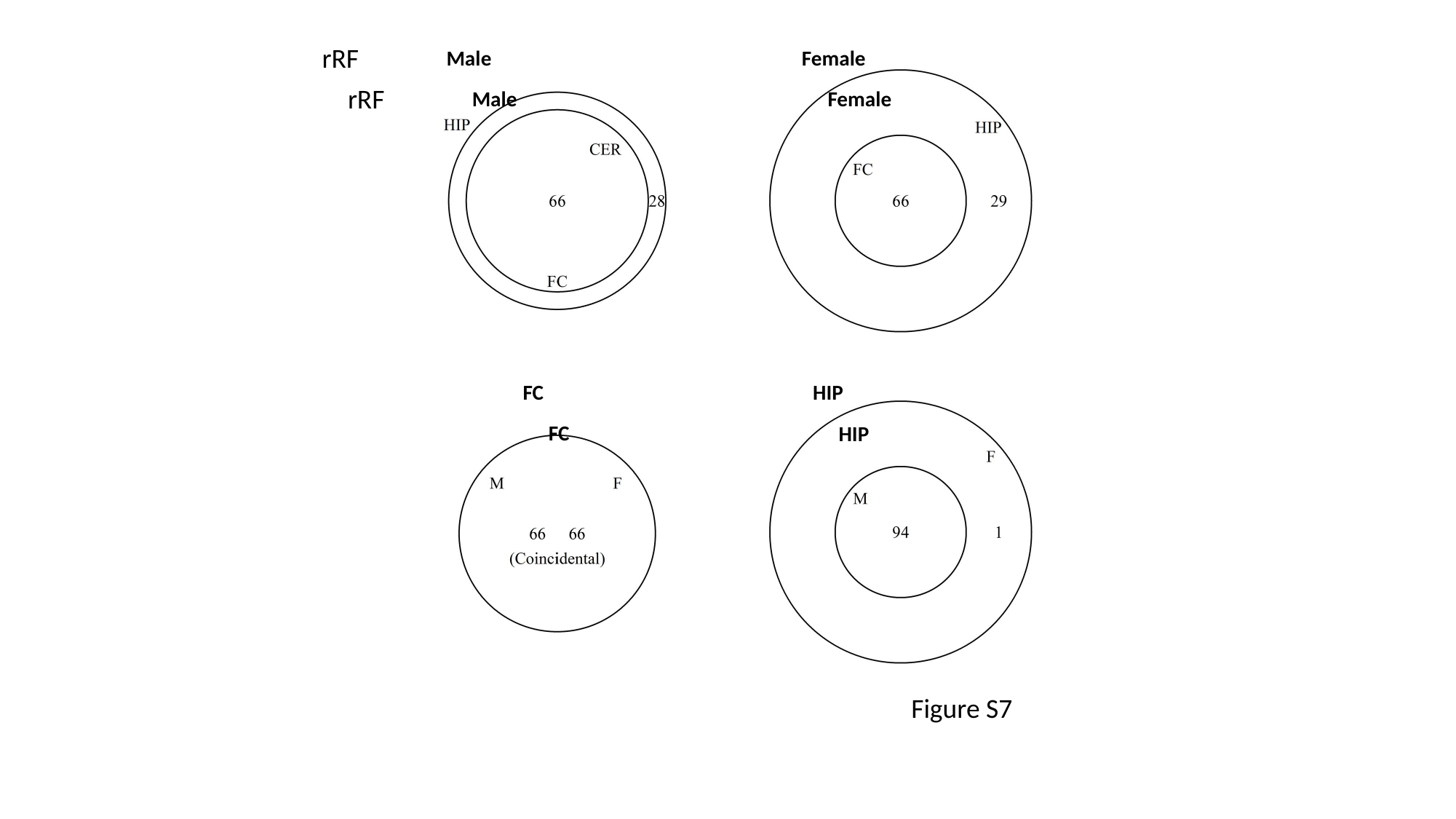

rRF
Male
Female
FC
HIP
Figure S7

## Slide 8
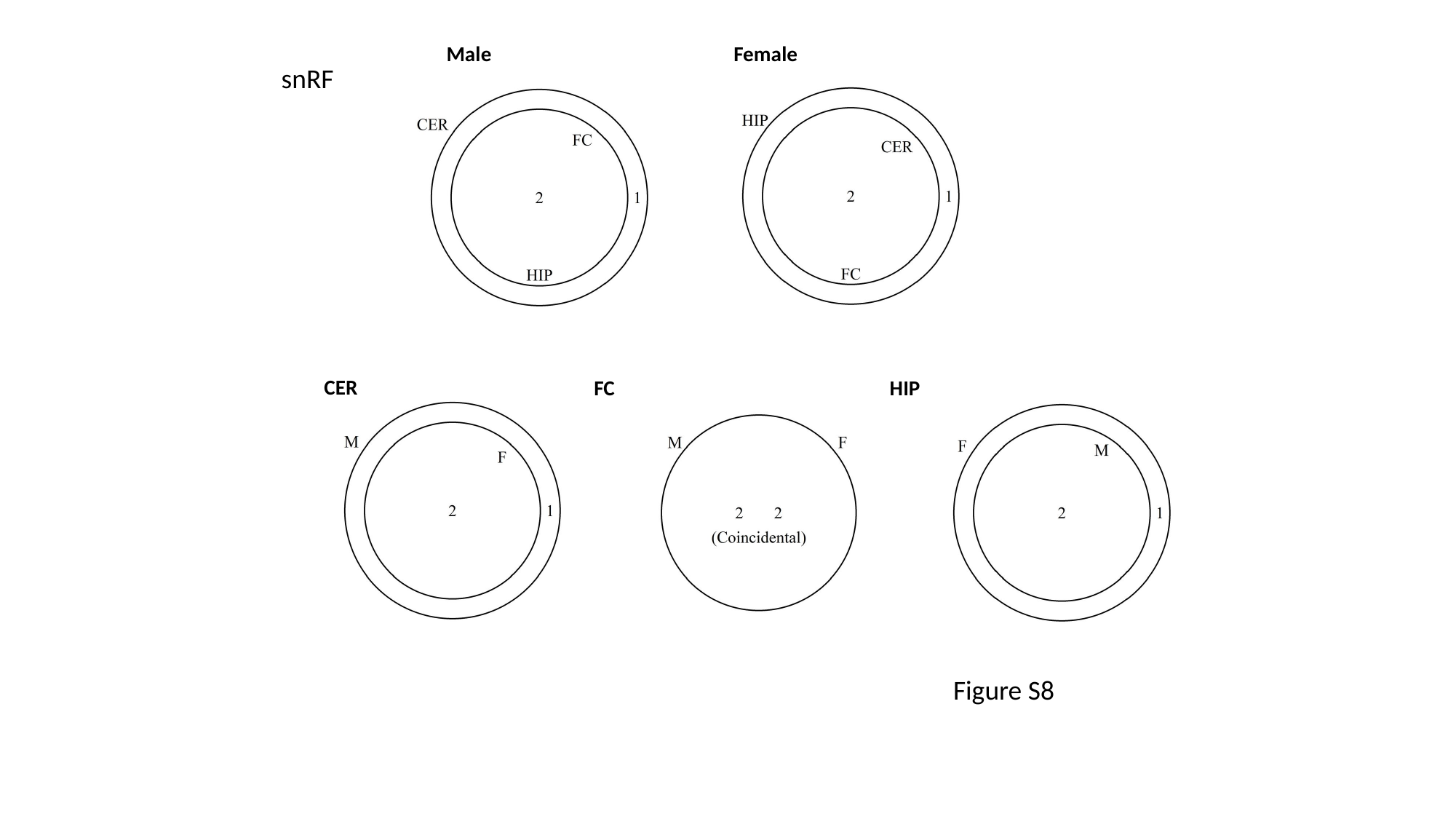

Male
Female
snRF
CER
FC
HIP
Figure S8
